# Risk factors for rapid axial length elongation with low concentration atropine for myopia control

**DOI:** 10.1038/s41598-021-88719-1

**Published:** 2021-06-03

**Authors:** Aicun Fu, Fiona Stapleton, Li Wei, Weiqun Wang, Bingxin Zhao, Kathleen Watt, Shiao Yu, Can Cui, Yong Lyu

**Affiliations:** 1 The First Affiliated Hospital of Zhengzhou University, No. 1 Jianshe road, Zhengzhou, 450000 China; 2 School of Optometry and Vision Science, UNSW, Sydney, 2052 Australia

**Keywords:** Diseases, Medical research, Risk factors

## Abstract

Three hundred and twenty-eight myopic children, randomized to use either 0.01% (N = 166) or 0.02% (N = 162) atropine were enrolled in this study. Gender, age, body mass index(BMI), parental myopia status, atropine concentration used, pupil diameter, amplitude of accommodation, spherical equivalent refractive error (SER), anterior chamber depth (ACD) and axial length (AL) were collected at baseline and 1 year after using atropine. Rapid AL elongation was defined as > 0.36 mm growth per year. Univariate analyses showed that children with rapid AL elongation tend to be younger, have a smaller BMI, use of 0.01% atropine, narrow ACD, lower SER, shorter AL, smaller change in pupil diameter between 1 year and baseline (all P < 0.05). Multivariate logistic regression analyses confirmed that rapid AL elongation was associated with children that were younger at baseline (*P* < 0.0001), use of 0.01% atropine (P = 0.04), a shorter baseline AL (P = 0.03) and a smaller change in pupil diameter between 1 year and baseline (*P* = 0.04). Younger children with shorter AL at baseline, less change in their pupil diameter with atropine treatment and using the lower of the two atropine concentrations may undergo rapid AL elongation over a 12 months myopia control treatment period.

## Introduction

The rising prevalence of myopia and the growing proportion of the population with high myopia result in significant economic and social impacts^1–3^.High myopia is associated with increased risk of visual impairment and blindness such as glaucoma, retinal detachment, choroidal neovascularization and myopic retinopathy^4^. These factors have generated research and clinical interest in the control of myopia progression.

Many studies have shown that low concentrations of atropine can control the progression of myopia in children with good efficacy, minimal side effects, convenient use, and slight rebound effects after discontinuation ^5–7^. Conversely, it has been observed that low concentration atropine has not effectively controlled myopia in all children^5–15^. Children with highly myopic parents were more likely to show rapid myopia progression with low concentration (0.01–0.05%) atropine^10^. Less initial myopia but not age, sex, and initial astigmatism was associated with less myopia progression in 0.05% atropine^8^. Younger age children showed faster myopic progression in 0.01% atropine. Studies have found a concentration-dependent response in myopia control with low concentration atropine^6,10,12,13^.

In the ATOM2 study^5,6^, the change in axial length (AL) and change in spherical equivalent refractive error (SER) were not synchronous, such that in the absence of change in SER, AL changed during use of 0.01% atropine. Conversely, a number of studies, including our previous study found that low concentration atropine reduced myopia progression not only through refractive changes, but also through control of axial elongation^10,12–15^. Meanwhile, myopia progression in children varies by race/ethnicity, and East/Southeast Asian children undergo more rapid myopia progression than white children^16,17^. The purpose of the current study was to specifically evaluate independent risk factors for rapid AL growth in myopic children using 0.01% and 0.02% atropine in mainland China, which have not previously been explored. Low dose atropine is widely used for myopia control in China but an appropriately powered RCT to explore independent risk factors has not been conducted.

## Results

**Figure 1:**
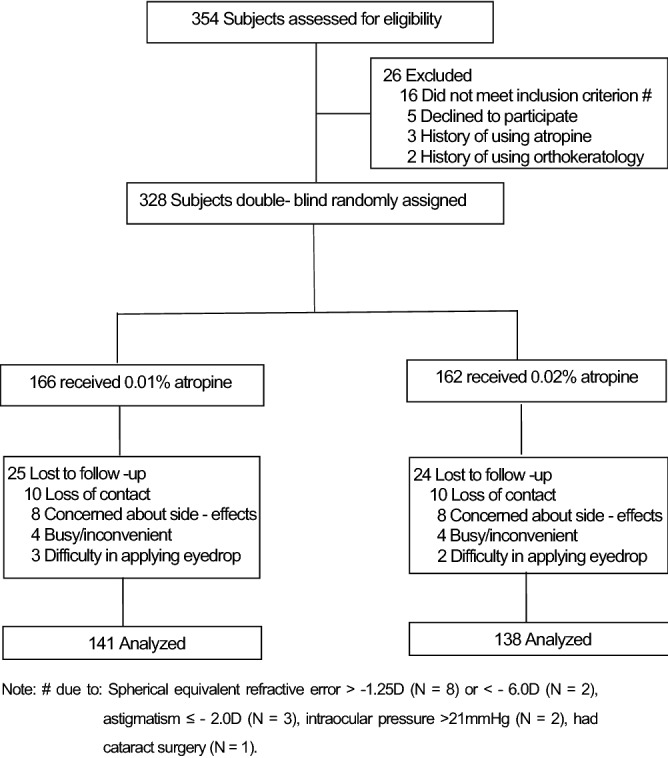
Subject recruitment and randomization flowchart.

A total of 328 children were enrolled into this cohort study, with 162 and 166 children in 0.02% atropine and 0.01% atropine groups, respectively (Fig. 1). No differences were found in age and other baseline parameters between groups (unpaired t-test, all P > 0.05). Table 1 showed a summary of the baseline data of the children grouped by atropine concentration and rate of AL change over the 12 months of the study. At 1-year follow-up, forty-nine children (14.9%) dropped out: 25 (15.1%) and 24 (14.8%) from the 0.01% atropine and 0.02% atropine, respectively (Fig. 1). No significant differences in baseline parameters were found between the children who discontinued and completed the study (unpaired t-test, all P > 0.05).

**Table 1:** Baseline characteristics of participants mean ± SD or n (%).

	Atropine concentration			Change in AL over 12 months (mm)		
	0.02% atropine	0.01% atropine	P-value	Rapid change in AL Mean ± SD, (min–max) 0.50 ± 0.16, (0.35 to 0.97)	Slower change in AL Mean ± SD, (min–max) 0.19 ± 0.12, (− 0.18 to 0.34)	P-value
N	138	141		132	147	
Age (year)	9.49 ± 1.83	9.39 ± 1.92	0.45	8.87 ± 1.69	10.76 ± 1.72	< 0.001
Body mass index (kg/m^2^)	17.58 ± 3.49	18.16 ± 3.24	0.33	17.33 ± 3.85	18.78 ± 3.37	< 0.001
Spherical equivalent refractive error (D)	− 2.79 ± 1.43	− 2.76 ± 1.61	0.32	− 2.27 ± 1.32	− 2.98 ± 1.68	< 0.001
Intraocular pressure (mmHg)	16.6 ± 3.6	17.0 ± 2.9	0.33	16.98 ± 2.78	16.58 ± 2.91	0.22
Pupil diameter (mm)	6.32 ± 0.66	6.15 ± 0.72	0.56	6.12 ± 0.76	6.07 ± 0.67	0.51
Accommodation amplitude (D)	15.20 ± 5.11	15.39 ± 4.32	0.29	14.87 ± 4.56	15.74 ± 5.25	0.22
Corneal astigmatism (D)	− 1.20 ± 0.48	− 1.22 ± 0.51	0.56	− 1.21 ± 0.58	− 1.24 ± 0.57	0.33
Corneal curvature (D)	42.82 ± 1.52	42.85 ± 1.41	0.69	42.81 ± 1.37	42.92 ± 1.45	0.66
Anterior chamber depth (mm)	3.64 ± 0.22	3.67 ± 0.19	0.76	3.60 ± 0.21	3.68 ± 0.27	< 0.001
Axial length (mm)	24.61 ± 0.72	24.59 ± 0.74	0.21	24.26 ± 0.81	24.68 ± 0.93	< 0.001
Outdoor activity (hours per day)^a^	2.53 ± 1.38	2.60 ± 1.32	0.67	2.52 ± 1.31	2.61 ± 1.35	0.21
Nearwork (hours per day)^#^	14.11 ± 2.01	14.23 ± 1.82	0.59	14.02 ± 2.82	14.22 ± 1.52	0.45
**Sex**						
Male	78	67	0.13	73 (55.3%)	72 (49.0%)	0.29
Female	60	74		59 (44.7%)	75 (51.0%)	
**Heredity**						
+ + (both parents myopic)	36	30		32 (24.2%)	34 (23.1%)	0.90
+ – (one parent myopic)	62	73	0.48	62 (47.0%)	73 (49.7%)	
−− (neither parent myopic)	40	32		38 (28.8%)	40 (27.2%)	

Rapid change in AL defined as > 0.36 mm/year.

^※^Outdoor activity = outdoor exercise + outdoor leisure activity.

^#^Nearwork = 3* (homework + reading + playing on cell phone) + 2* (using computer + playing video game) + 1* (watching TV).

The mean ± SD change in AL over the 12 months of the study was 0.35 ± 0.24 mm. The mean ± SD (min–max) change AL in rapid growth group and slower growth group was 0.50 ± 0.16 mm (0.35 to 0.97) and 0.19 ± 0.12 mm (-0.18 to 0.34), respectively. AL change was 0.30 ± 0.22 mm and 0.36 ± 0.19 mm in the 0.02% and 0.01% atropine, respectively (P = 0.02). One hundred twenty eight (90.8%) and 124 children (89.9%) in the two corresponding atropine groups who completed the 1-year follow-up had good compliance. Based on the univariate analysis (Table 2), we found that younger age, smaller BMI, both parents myopic, using lower concentration atropine, narrow ACD, lower SER, shorter AL, a smaller change pupil diameter from baseline were the risk factors for a rapid change in AL (all P < 0.05). There were, however, no statistically significant associations with gender, one parent myopic, baseline IOP, pupil diameter and AMP, corneal curvature, corneal astigmatism and change in AMP between 1 year and baseline.

**Table 2:** Univariate and multivariate logistic regression analyses showing risk factors associated with rapid axial length elongation over 12 months.

Risk factors	Non-adjusted OR, (95% CI)	P value	Adjust OR, (95% CI)	P value
**Gender**				
Male	Reference		Reference	
Female	0.78 (0.48–1.08)	0.22	0.79 (0.41−1.17)	0.73
Age (years)	0.54 (0.44–0.64)	** < 0.0001**	0.58 (0.42–0.74)	** < 0. 0001 **^**a**^
Body mass index (kg/m^2^)	0.87 (0.82–0.92)	**0.0006**	0.90 (0.71−1.09)	0.63
**Heredity**				
−− (neither parent myopic)	Reference		Reference	
+ − (one parent myopic)	0.59 (0.16–1.02)	0.12	0.62 (0.20–1.04)	0.25
+ + (both parents myopic)	0.57 (0.19–0.95)	**0.04**	0.60 (0.18–1.02)	0.35
**Atropine concentration**				
0.01%	Reference		Reference	
0.02%	0.74 (0.53–0.95)	**0.044**	0.67 (0.38–0.96)	**0.04 **^**b**^
Intraocular pressure (mmHg)	1.06 (0.96–1.16)	0.22	1.05 (0.95–1.15)	0.65
Pupil diameter (mm)	1.07 (0.72–1.42)	0.86	0.84 (0.53–1.15)	0.74
Accommodation amplitude (D)	0.95 (0.88–1.02)	0.08	0.99 (0.85–1.13)	0.36
Corneal curvature (D)	0.86 (0.70–1.02)	0.16	0.84 (0.58–1.10)	0.21
Corneal astigmatism (D)	0.62 (0.18–1.06)	0.32	0.58 (0.12–1.04)	0.52
Anterior chamber depth (mm)	3.88 (1.34–6.42)	**0.01**	1.15 (0.22–2.08)	0.93
Spherical equivalent refractive error (D)	1.34 (1.13–1.55)	**0.0003**	1.02 (0.73–1.31)	0.65
AL baseline (mm)	0.60 (0.44–0.76)	**0.0001**	0.46 (0.21–0.71)	**0.03 **^**c**^
Outdoor activity (hours per day)^※^	0.84 (0.65–1.03)	0.11	0.93 (0.71–1.15)	0.52
Nearwork (hours per day)^#^	0.75 (0.42–1.08)	0.35	0.82 (0.60−1.04)	0.33
Change in pupil diameter between 1 year and baseline (mm)	0.56 (0.37–0.75)	**0.03**	0.46 (0.24–0.68)	**0.04 **^**d**^
Change in accommodation amplitude between 1 year and baseline (D)	0.58 (0.12–1.04)	0.21	0.60 (0.20–1.00)	0.49

The bold represents P value less than 0.05.

Note: # due to: Spherical equivalent refractive error > -1.25D (N = 8) or <—6.0D (N = 2), astigmatism ≤—2.0D (N = 3), intraocular pressure > 21 mmHg (N = 2), had cataract surgery (N = 1).

*OR* odds ratio; *CI* confidence interval.

^a^Adjusted: body mass index (BMI), spherical equivalent refractive error (SER), accommodation amplitude (AMP), anterior chamber depth (ACD), outdoor activity and nearwork.

^b^Adjusted: age, parental myopia status, SER, AMP, corneal curvature, ACD and nearwork.

^c^Adjusted: gender, age, BMI, SER, AMP, corneal curvature, ACD, outdoor activity and nearwork.

^d^Adjusted: age, BMI, SER, AMP, pupil diameter, corneal curvature, ACD and nearwork.

^※^Outdoor activity = outdoor exercise + outdoor leisure activity. # Nearwork = 3* (homework + reading + playing on cell phone) + 2* (using computer + playing video game) + 1* (watching TV).

Multivariate logistic regression analyses after adjusting for potential confounders showed that the factors associated with rapid AL elongation children were younger age (OR = 0.58, 95% CI 0.42–0.74, P < 0.0001), using lower concentration atropine (OR = 0.67, 95% CI 0.38–0.96, P = 0.04) and shorter AL at baseline (OR = 0. 46, 95% CI 0.21–0.71, P = 0.03 ), lower change pupil diameter between 1 year and baseline (OR = 0.46, 95% CI 0.24–0.68, P = 0.04) (Table 2). There was a 42% higher risk of rapid AL elongation with every year of decreasing age at baseline. Meanwhile, the risk of rapid AL elongation using 0.01% atropine increased by 33% in comparison with 0.02% atropine. Similarly, the risk of rapid AL elongation increased by 54% for every 1 mm shorter AL at baseline and 54% for every 1 mm lower change in pupil diameter over the 12 months of the study.

## Discussion

In this prospective study, the axial length of the younger children, using lower concentration atropine, with shorter axial length at baseline and smaller change in pupil diameter may still increase rapidly while receiving atropine treatment in mainland China. These factors seem to suggest that these children may be less responsive to the effects of low concentration atropine.

Rapid AL elongation despite the use of low concentration atropine treatment has been described in other different atropine concentrations and populations studies. In the ATOM2 study^5,6^, where children were randomized to atropine concentrations of 0.01%, 0.1% and 0.5%, the AL change after twelve months was 0.24 ± 0.19 mm, 0.13 ± 0.18 mm and 0.11 ± 0.17 mm, respectively. In a study of 0.01%, 0.025% and 0.05% atropine concentrations, Yam et al.^12,15^ found that the AL change after 1 year was 0.36 ± 0.29 mm, 0.29 ± 0.20 mm and 0.20 ± 0.25 mm, after 2 years was 0.59 ± 0.38 mm, 0.50 ± 0.33 mm, and 0.39 ± 0.35 mm, respectively. In a one year study of Korean myopic children^10^, the AL elongation was about 0.44 ± 0.32 mm, 0.30 ± 0.24 mm and 0.23 ± 0.25 mm in 0.01%, 0.025% and 0.05% atropine, respectively. In a study of Chinese myopic children^14^, the AL elongation after one year was about 0.32 ± 0.19 mm and 0.41 ± 0.09 mm in 0.01% atropine and placebo, respectively. By comparison, in the present study the AL elongation was 0.36 ± 0.19 mm with 0.01% and 0.30 ± 0.22 mm with 0.02% atropine, 0.02% atropine had a better effect on myopia control than 0.01% atropine^13^. There seems to be considerable variation in the range of AL change results with some children changing very little and others increasing very quickly. Other reports of low concentration atropine for controlling myopia progression in children measured refraction and not AL^8,9,11,18^. They defined myopia progression of more than 0.50D or 1.0D at 1 year as rapid myopic progression. Again rates of refractive progression varied considerably between studies, risk factors for faster myopic progression in low concentration atropine studies include younger age at baseline^11,18^, higher initial myopia^8^, and a family history of high myopia^10^. This confirms the importance of conducting appropriately controlled studies to explore independent risk factors associated with rapid AL elongation in myopic children in mainland China treated with low concentration atropine.

In the current study, baseline age had a significantly negative correlation with AL increase in myopic children using 0.01% or 0.02% atropine. The younger children were at baseline, the more the AL elongation was evident at the end of the study period. This was consistent with three other studies which explored the same relationship between baseline age and AL increase in myopic children using atropine and whose baseline profiles were similar to the current study. Joachimsen et al.^11^, Wei et al.^14^ and Lee et al.^18^ found that younger children in German, mainland China and Taiwan may still progress quickly while using 0.01%, 0.01% and 0.05% atropine, respectively. It is well established that there is slowing of physiological change in AL in older children^19^. Conversely, Moon et al.^10^ and Wu et al.^8^ reported no relationship between baseline age and AL change in Taiwanese children using 0.05% atropine and in Korean children using 0.01%, 0.025% or 0.05% atropine. These contrasting results may arise due to different study populations, different baseline characteristics and methodological and analytical differences.

We also found that the children with 0.01% atropine had a more rapid AL elongation and a worse myopic control effect than 0.02% atropine^13^. Other reports have also found a concentration-dependent response in myopia control with low concentration atropine^6,10,12,14^. Side effects and adverse effects are reportedly similar in 0.01% and 0.02% atropine^13,20^, suggesting that if children using 0.01% atropine are not achieving adequate myopia control, changing to the 0.02% concentration could be considered. It is important to use an individualized atropine concentration to control myopia development.

The children with a smaller change in pupil diameter from baseline to 1 year had rapid AL elongation in our study. The underlying reasons why these children did not respond well are unclear. So far, it is unclear how low concentration atropine acts to inhibit myopia progression. One theory^21^ suggests that increased ultraviolet exposure (secondary to pupil dilation) may increase collagen cross-linking within the sclera, thereby limiting scleral growth during myopia progression and AL elongation. The greater change in pupil diameter, may be due to the better absorption of the drug, greater collagen cross-linking within the sclera and a superior effect on controlling myopia progression. Recently, studies have shown that adding 0.01% atropine eye drops to OK therapy was more effective than OK alone for controlling myopia progression in children^22,23^. One of the possible mechanisms may be low concentration atropine’s mydriatic effect, as other studies have shown that myopia control effect of OK lens was also affected by pupil diameter^23,25^. Children with larger pupil diameter would receive a greater proportion of peripheral myopic defocus associated with OK lens wear, which in turn may be relate to better myopia control^23^. This is a possible mechanism that warrants further investigation.

We found that a rapid AL elongation related significantly to shorter AL at baseline, but not to initial levels of myopia. In other words, the shorter AL at baseline, the more the AL elongation was found with low concentration atropine, irrespective of baseline refractive error. Lin^26^ however found that highly myopic children had less increase in AL after using 0.125% atropine. Wu et al.^8^ conversely found that higher initial myopia corresponded significantly to a higher chance of myopia progression irrespective of atropine concentration. Moon et al.^10^ found that baseline refraction and AL were not associated with the rate of myopia progression irrespective of atropine concentration, however only age, family history and AL were selected for inclusion in the multivariate logistic regression analyses of this study. Several studies^27,28^ have established a strong association between the eye’s AL and its refractive error. Myopia progression usually occurs due to excessive AL elongation of the eye, as evidenced by the strong association between changes in myopia progression and changes in AL growth^19,29,30^. There was a strong association between the change in AL and change in SER after 1-year treatment in the present study. Another study^14^ found that 0.01% atropine reduced myopia progression by 34.2% and axial elongation by 22.0% compared with the placebo group. Howerver, in the ATOM2 study^6^, while the degree of myopia was stable, the AL continued to increase between 8 and 24 months after using 0.01% atropine. For assessing the efficacy of interventions to control the progression of myopia in children, we recommend that the change in AL is not be used interchangeably with the change in refraction.

This study did not consider the rate of change in AL using age matched emmetropes, however previous studies^31,32^ describing the growth curve for AL with age showed that the rate of AL elongation in emmetropes was slower than in myopes, and the AL growth slowed down in emmetropes as the children became older, but little or no decrease in the rate of change of AL in myopic children is observed. Other limitations were that the study measured axial elongation over a 1 year period only and the potential bias introduced by loss to follow-up of approximately 15% in each low concentration atropine group. Further investigations including age matched emmetropes and using a study design with a longer follow-up time are required to confirm the present findings. An additional limitation of the present study, is that the age at onset of myopia was not available. Previous studies^33,34^ have identified that age at onset of myopia is a risk factor for myopia progression and this factor should included in future studies.

In conclusion, our study showed that the axial length in young children, using lower concentration of atropine, having a shorter axial length at baseline and smaller change in pupil diameter may still increase rapidly while receiving low concentration atropine treatment in mainland China. If the axial length of these children with these characteristics continues to grow at a rate similar to that before the start of atropine treatment, then a change in atropine concentration or combining with other myopia control therapies to control the progression of axial length may be considered.

## Methods

We report a prospective doubled blinded randomized controlled trial of 0.01% and 0.02% atropine in myopic children recruited from the First Affiliated Hospital of Zhengzhou University. This study was part of a larger series of clinical studies which also evaluated a further three non-contemporaneous non randomized patient groups, not reported here, including subjects wearing single-vision spectacles, subjects with low myopia using 0.005% atropine, and a subsequent group evaluating 0.02% atropine used every other day.

In this prospective study, three hundred and twenty-eight Chinese myopic children (right eyes, Han nationality) who presented between July 2016 and October 2017 were recruited. The inclusion criteria were: 6–14 years of age, cycloplegic autorefraction SER from − 6.00 D to − 1.25 D, astigmatism less than 2.0 D, anisometropia of less than 1.0 D, monocular best corrected visual acuity of 16/20 or better, intraocular pressures (IOP) between 10 and 21 mmHg, no other eye diseases and surgery, no ocular and systemic conditions that might affect vision or vision development. Exclusion criteria were: previous use of atropine, pirenzepine, soft contact lenses, rigid gas permeable and orthokeratology contact lens to control myopia progression; inability to comply with the study visit schedule. This study was approved by the Medical Ethics committee of the First Affiliated Hospital of Zhengzhou University and registered in the Chinese Clinical Trial Registry (registration number: ChiCTR-IPD-16008844, first registration in 14/07/2016). This study conformed to the tenets of the Declaration of Helsinki. Written informed consent was obtained from parents before the procedures, and possible risks were fully explained before treatment initiation.

The 0.01% and 0.02% atropine eye drops (pH value of 5.4–5.6, 3-mL sealed bottle, kept away from the light, 15–25 ℃ room temperature storage, discarded one month after opening the bottle) were made by diluting 1% atropine (Eye & ENT Hospital, Affiliated to Fudan University) with saline under sterile conditions, with the addition of the preservative (0.3 mg/ml ethylparaben). Subjects were double-blindly and randomly assigned to 0.01% and 0.02% concentration, where random numbers were generated by computer algorithm. The children were prescribed constant wear of full correction single-vision spectacle lenses (SV) with the highest positive/least negative power consistent with optimum visual acuity and with one drop of 0.01% or 0.02% atropine eye drops administered into both eyes once nightly before bed time.

Corneal power, anterior chamber depth (ACD) and AL were evaluated using a non-contact partial coherence interferometer (IOLMaster; Carl Zeiss Meditec AG, Germany). On each occasion, five successive measurements were taken and their mean was used as a representative value. Based on other two similar studies^35,36^, rapid AL elongation was defined as > 0.36 mm growth per year (i.e., equivalent myopic progression 1.00 D per year) and children were categorized as either having rapid or slow AL elongation.Outdoor activity time (hours per day) and nearwork time (hours per day)^12^ were assessed using a paper questionnaire. Cycloplegic autorefraction was performed after the instillation of four drops of compound tropicamide eye drops^37,38^ (0.5% tropicamide and 0.5% neo-synephrine) (Santen, Japan) administered 10 min apart in each of the patients’ eyes. Ten minutes after the instillation of the fourth drop, three autorefraction measurements were taken (Topcon RM 8000A, CA) and a mean was obtained. The degree of myopia was expressed as SER.

Children were provided with four bottles of eye drops after the first examination and subsequent each follow-up. When the child was reexamined, they were asked to return all four bottles and compliance was assessed according to the remaining amount of eye drops. One-drop of eye drops is about 0.04 ml, one child will use more than 2.4 ml each month. If a child's remaining eye drops in any bottle exceeded 10% (about 1 ml) of the total amount of each bottle, then his compliance was not good. In order to improve compliance, we have also adopted two methods: 1. we explained to children and their parents the importance of using eye drops correctly every day for myopia control after they entered the research group. 2. The WeChat group was set up for all the children’s parents in the research group, there were two colleagues of the research group answered all kinds of questions encountered by the children in the medication process.

Continuous baseline variables were expressed as mean ± standard deviation (SD) and evaluated by using Student *t*-test. Categorical variables, such as gender, atropine concentration and parental myopia status, were expressed as percentage (%) and evaluated by the Chi-squared test. Univariate analysis and multivariate logistic regression analyses after adjusting for covariates were used to determine risk factors for rapid AL elongation, whether or not the covariates was adjusted was determined by the following principle: when a factor was added to this model, the matched odds ratio changed by at least 10%. A value of P < 0.05 was considered statistically significant. All analyses were performed using Empower (R) (www. empowerstats.com, X & Y solutions Inc., Boston, MA) and R (http://www.R-project.org).

## References

[1] Lim MCC, Gazzard G, Sim EL, Tong L, Saw SM (2009). Direct costs of myopia in singapore. Eye.

[2] Holden BA (2016). Global prevalence of myopia and high myopia and temporal trends from 2000 through 2050. Ophthalmol..

[3] Smith TST (2009). Potential lost productivity resulting from the global burden of uncorrected refractive error. Bull World Health Org..

[4] Wong TY (2014). Epidemiology and disease burden of pathologic myopia and myopic choroidal neovascularization: An evidence-based systematic review. Am. J. Ophthalmol..

[5] Chia A (2014). Atropine for the treatment of childhood myopia: changes after stopping atropine 0.01%, 0.1% and 0.5%. Am. J. Ophthalmol..

[6] Chia A, Lu QS, Tan D (2016). Five-year clinical trial on atropine for the treatment of myopia 2: myopia control with atropine 0.01% eye drops. Ophthalmol..

[7] Sacchi M (2019). Efficacy of atropine 0.01% for the treatment of childhood myopia in European patients. Acta Ophthalmol..

[8] Wu PC, Yang YH, Fang PC (2011). The long-term results of using low-concentration atropine eye drops for controlling myopia progression in schoolchildren. J. Ocul. Pharmacol. Ther..

[9] Clark TY, Clark RA (2015). Atropine 0.01% Eye drops significantly reduce the progression of childhood myopia. J. Ocul. Pharmacol. Ther..

[10] Moon JS, Shin SY (2018). The diluted atropine for inhibition of myopia progression in Korean children. Int. J. Ophthalmol..

[11] Joachimsen L (2019). A pilot study on the efficacy and safety of 0.01% atropine in German schoolchildren with progressive myopia. Ophthalmol. Ther..

[12] Yam JC (2019). Low concentration atropine for myopia progression (LAMP) study: A randomized, double-blinded, placebo controlled trial of 0.05%, 0.025%, and 0.01% atropine eye drops in myopia control. Ophthalmol..

[13] Fu AC (2020). Effect of low-dose atropine on myopia progression, pupil diameter and accommodative amplitude: Low-dose atropine and myopia progression. Br. J. Ophthalmol..

[14] Wei SF (2020). Safety and efficacy of low-dose atropine eyedrops for the treatment of myopia progression in chinese children: A randomized clinical trial. JAMA Ophthalmol..

[15] Yam JC (2020). Two-year clinical trial of the low-concentration atropine for myopia progression (LAMP) study: Phase 2 report. Ophthalmol..

[16] Donovan L (2012). Myopia progression rates in urban children wearing single-vision. Optom. Vis. Sci..

[17] Luong TQ (2020). Racial and ethnic differences in myopia progression in a large, diverse cohort of pediatric patients. Invest. Ophthalmo. Vis. Sci..

[18] Lee JJ (2006). Prevention of myopia progression with 005% atropine solution. J. Ocul. Pharmacol. Ther..

[19] Diez PS (2019). Growth curves of myopia-related parameters to clinically monitor the refractive development in Chinese schoolchildren. Graefes Arch. Clin. Exp. Ophthalmol..

[20] Cooper J, Eisenberg N, Schulman E, Wang FM (2013). Maximum atropine dose without clinical signs or symptoms. Optom. Vis. Sci..

[21] Prepas SB (2008). Light, literacy and the absence of ultraviolet radiation in the development of myopia. Med. Hypotheses..

[22] Kinoshita N (2018). Additive effects of orthokeratology and atropine 0.01% ophthalmic solution in slowing axial elongation in children with myopia: first year results. Jpn. J. Ophthalmol..

[23] Kinoshita N (2020). Efficacy of combined orthokeratology and 0.01% atropine solution for slowing axial elongation in children with myopia: A 2-year randomized trial. Sci. Rep..

[24] Chen Z (2012). Impact of pupil diameter on axial growth in orthokeratology. Optom. Vis. Sci..

[25] Santodomingo-Rubido J, Villa-Collar C, Gilmartin B, Gutiérrez-Ortega R (2013). Factors preventing myopia progression with orthokeratology correction. Optom. Vis. Sci..

[26] Lin HJ (2014). Overnight orthokeratology is comparable with atropine in controlling myopia. BMC Ophthalmol..

[27] Richter GM (2017). Ocular determinants of refractive error and its age and sex-related variations in the Chinese American eye study. JAMA Ophthalmol..

[28] Hou W (2018). Axial elongation in myopic children and its association with myopia progression in the correction of myopia evaluation trial. Eye Contact Lens..

[29] Gwiazda J (2003). A randomized clinical trial of progressive addition lenses versus single vision lenses on the progression of myopia in children. Invest. Ophthalmol. Vis. Sci..

[30] Saw SM (2005). Eye growth changes in myopic children in Singapore. Br. J. Ophthalmol..

[31] Jones LA (2005). Comparison of ocular component growth curves among refractive error groups in children. Invest. Ophthalmol. Vis. Sci..

[32] Wong HB, Machin D, Tan SB, Wong TY, Saw SM (2010). Ocular component growth curves among Singaporean children with different refractive error status. Invest. Ophthalmol. Vis. Sci..

[33] Chua SYL (2016). Age of onset of myopia predicts risk of high myopia in later childhood in myopic Singapore children. Ophthal. Physiol. Opt..

[34] Saw SM (2005). Incidence and progression of myopia in singaporean school children. Invest. Ophthalmol. Vis. Sci..

[35] Cho P, Cheung SW (2012). Retardation of myopia in orthokeratology (ROMIO) study: A 2-year randomized clinical trial. Invest. Ophthalmol. Vis. Sci..

[36] Cho P, Cheung SW (2017). Protective role of orthokeratology in reducing risk of rapid axial elongation: A reanalysis of data from the ROMIO and TO-SEE studies. Invest. Ophthalmol. Vis. Sci..

[37] Lin LL (1998). The cycloplegic effects of cyclopentolate and tropicamide on myopic children. J. Ocul. Pharmacol. Ther..

[38] Yazdani N, Sadeghi R, Momeni-Moghaddam H, Zarifmahmoudi L, Ehsaei A (2018). Comparison of cyclopentolate versus tropicamide cycloplegia: A systematic review and meta-analysis. J. Optom..

